# Comparative Study of Spinal, Epidural, and Sequential Combined Spinal Epidural Anesthesia in Geriatric Patients for Transurethral Resection of the Prostate

**DOI:** 10.7759/cureus.58099

**Published:** 2024-04-12

**Authors:** Arvind Tomar, Jay Brijesh Singh Yadav, Dheer Singh, Rakesh Bahadur Singh, Reena Rani Verma, Sarika Pandey

**Affiliations:** 1 Anesthesiology, Uttar Pradesh University of Medical Sciences, Saifai, IND; 2 Physiology, SVS Medical College, Mahabubnagar, IND

**Keywords:** turp, geriatric, sequential, epidural, spinal

## Abstract

Background: Sequential combined spinal epidural anesthesia (CSEA) is probably the greatest advancement in the central neuraxial block in this decade for geriatric patients due to the potential advantages of both spinal and epidural anesthesia. This study was designed to compare the clinical effects of sequential CSEA versus spinal and epidural anesthesia in geriatric patients undergoing transurethral resection of the prostate (TURP).

Methods: Ninety patients aged 65 to 80 years were randomly allocated into three groups of 30 each. Group A (n=30) patients were administered spinal anesthesia with 2.5 ml of 0.5% hyperbaric bupivacaine, group B (n=30) received epidural anesthesia with 15 ml of 0.5% isobaric bupivacaine, and group C (n=30) received sequential CSEA with 1 ml of 0.5% hyperbaric bupivacaine and 6 ml of 0.5% isobaric bupivacaine given through epidural route to extend the block up to T10. Patients were observed for hemodynamic parameters, sensory and motor block, total dose required to establish the desired level, and patient satisfaction score.

Results: None of the patients were excluded in the study. Group A patients reported rapid onset of sensory block (3.08±11.57 minutes) compared to group B (11.57±1.48 minutes), and group C (5.47±1.25 minutes). The onset of motor block was expeditious in group A (8.08±1.0 minutes) compared to group B (20.33±1.86 minutes) and group C (15.53±1.31 minutes). Patients in group B had maximum hemodynamic stability but with delayed onset and were technically more complex than group A. Patients in group C were hemodynamically more stable than group A. They had a faster onset of action with decreased doses of local anesthetic drug required compared to group B.

Conclusion: Sequential CSEA is a safe, effective, and reliable technique that combines the advantages of both spinal and epidural while minimizing their disadvantages. It has the advantage of stable hemodynamic parameters along with the provision of prolongation analgesia for geriatric patients undergoing TURP surgery.

## Introduction

Benign Prostatic Hyperplasia is the most common cause of bladder outflow obstruction in men older than 60 years [1]. Transurethral resection of the prostate (TURP) has been used for surgical intervention in mild to moderate-size BPH [2,3]. In developing countries where the availability of high-power lasers is limited due to financial constraints, TURP has stood the test of challenge by many modalities and has been the gold standard in prostate surgery for decades [4]. Skillful application of regional anesthesia techniques i.e., spinal, epidural, and combined spinal-epidural anesthesia (CSEA) broadens the anesthetist’s range of options. It provides optimal anesthetic care which includes postoperative analgesia, improved patient satisfaction, and early recognition of fatal complications like bladder perforation, capsular tear, and TURP syndrome while general anesthesia is preferred in patients when regional anesthesia is contraindicated [5]. Amongst the regional anesthetic techniques, spinal anesthesia is regarded as the technique of choice for TURP [6]. It offers a rapid onset of action, provides reliable surgical anesthesia, good muscle relaxation, requires a small amount of anesthetic agent, and is comparatively simple to perform. When performed under epidural anesthesia, there are fewer incidences of cardiovascular instability and post-dural puncture headaches compared to spinal anesthesia, and it works well for longer surgical procedures [7]. CSEA has the advantage of attenuating the neurohumoral stress response to surgery, producing vasodilatation thereby reducing afterload, reducing the incidence of a thromboembolic phenomenon, avoiding polypharmacy, and enhancing early ambulation [8,9]. Another important technique is called sequential CSEA (SCSEA), which is commonly used nowadays in high-risk elderly patients posted for major orthopedic and gynecological surgeries [10,11]. In this technique, the lower dose of the drug in the subarachnoid block, inadequate for surgery is used and the height of the block is deliberately extended cephalad with the epidural drug. Fewer studies compared the effects of sequential CSEA versus spinal anesthesia in orthopedic surgery [12]. Hence, we aimed to compare the spinal, epidural, and sequential CSEA in terms of hemodynamic stability, sensory and motor blockade, dose requirement, and complications in TURP.

Primary objective

The aim of this study is to compare hemodynamic effects in spinal, epidural, and sequential CSEA in geriatric patients for TURP.

Secondary objectives

The secondary objectives are 1. hemodynamic variables like heart rate (HR), systolic blood pressure (SBP), diastolic blood pressure (DBP), mean arterial pressure (MAP), respiratory rate (RR), and oxygen saturation (SpO_2_); 2. onset and level of sensory and motor block; 3. total dose (milligram) of bupivacaine required to establish the desired level of block; 4. patient satisfaction score; and 5. complications, if any.

## Materials and methods

After obtaining approval from the Uttar Pradesh University of Medical Sciences (UPUMS) Institutional Ethics Committee (1660/UPUMS/Dean/2019-20/E.C 56175/2019-18) and informed consent of the patients, this study was conducted in the Department of Anesthesia, Uttar Pradesh University of Medical Sciences in Saifai, India. The study was conducted in a prospective randomized double-blind manner.

Inclusion and exclusion criteria

Patients aged 65-80 years with American Society of Anesthesiologists (ASA) physical status classifications I and II, and duration of surgery less than 90 minutes were included in the study. Patients who did not consent, had local anesthesia allergies, a history of substance abuse, and bleeding disorders were excluded from the study.

Sample size calculation and randomization

After discussion with a statistician and based on previous studies [12], considering an alpha error of 0.05 and power of study as 95%, the estimated sample size comes out to be 30 patients per group.

Patients were randomly assigned into three groups of 30 each, employing a computer-generated random number table. Group A (n=30) received spinal anesthesia, group B (n=30) received epidural anesthesia, and group C (n=30) received SCSEA.

Methodology

The pre-anesthetic check-up was done for all the patients, details related to clinical history and general physical examination were recorded and all necessary investigations were carried out.

All patients were kept nil per oral six hours before surgery. Upon arrival in the operation room, ASA standard monitoring devices were attached and electrocardiography (ECG), non-invasive blood pressure, peripheral oxygen saturation (SpO_2_), and temperature monitoring were carried out and baseline parameters were recorded. Intravenous access was obtained with an 18G cannula and patients were preloaded with lactated ringer`s solution at 20 ml.kg-1 body weight. The patients were positioned in a sitting or left lateral position. After aseptic preparation and draping of the lumbar area patients were administered regional anesthesia technique at L3-L4 level according to the assigned group.

Group A (n=30) received spinal anesthesia with 2.5 ml of 0.5% hyperbaric bupivacaine, group B (n=30) received epidural anesthesia with 15 ml of 0.5% isobaric bupivacaine, and group C (n=30) received sequential CSEA with 1 ml of 0.5% hyperbaric bupivacaine using a combined spinal epidural needle and 6 ml of 0.5% isobaric bupivacaine administered through the epidural route to extend the block up to T10.

After the procedure, patients were placed in a lithotomy position and oxygen was administered at 5-6 liters per minute via face mask. Monitoring of hemodynamic parameters such as HR, SBP, DBP, MAP, SpO_2_, and ECG was carried out every five minutes till the end of surgery. Onset, duration and degree of sensory blockade and onset, duration and degree of motor blockade were recorded. The sensory block was assessed by the pinprick method every minute along the midclavicular line bilaterally. The degree of sensory block was assessed bilaterally according to Gromley and Hill grade: 0=normal sensation, 1=blunted sensation, 2=no sensation. The degree of motor block was assessed bilaterally using a modified Bromage scale 0=no motor block, 1=can flex knee, move foot, but cannot raise leg, 2=can move foot only, 3=cannot move foot or knee. Surgery was started when the sensory level reached to T10 level and the modified Bromage score of 3 was achieved. The total dose of bupivacaine required to establish the desired block level and prolong the block was noted. Complications such as hypotension (SBP<90 mmHg) were treated with mephetermine 6 mg iv in incremental doses. Duration of analgesia and any adverse effects were noted. The satisfaction of patients was assessed using a Likert verbal rating scale immediately after surgery with 1. extremely dissatisfied; 2. dissatisfied; 3. somewhat dissatisfied; 4. undecided; 5. somewhat satisfied; 6. satisfied; 7. extremely satisfied after the completion of the surgery, patients were shifted to the post-anesthesia care unit.

Statistical analysis

The quantitative variables are expressed as mean±SD and compared between groups using unpaired t-test and within groups across follow-up using paired t-test. Qualitative variables were compared between groups using the chi-square test. A p-value <0.05 is considered statistically significant. The data was stored in an MS Excel spreadsheet and statistical analysis was performed using IBM SPSS Statistics for Windows, Version 20 (Released 2011; IBM Corp., Armonk, New York, United States).

## Results

**Figure 1:**
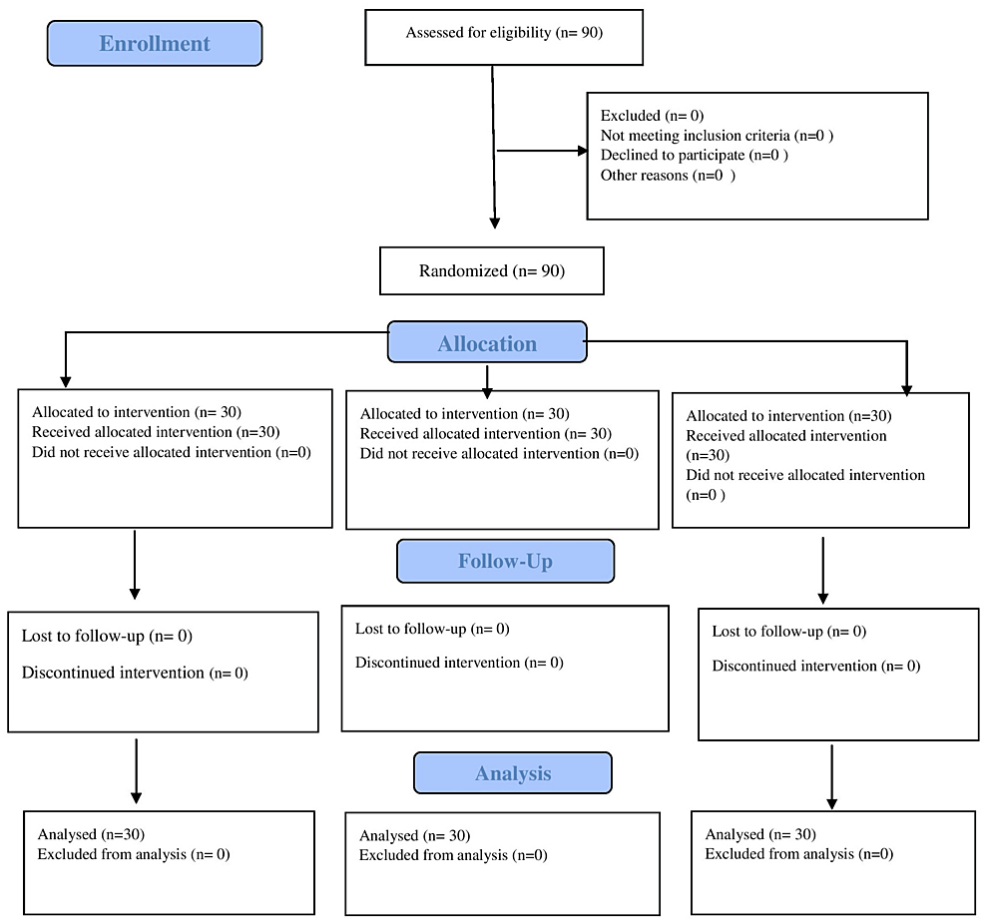
CONSORT flow diagram

None of the patients were excluded in our study. The study population comprised of 90 patients undergoing surgery for BPH were allocated into three groups of 30 patients each, were enrolled for the study (Figure 1).

Table 1 shows the distribution of age between the groups. The mean age of the patients of group A, group B, and group C was 72.30±5.75, 70.90±4.53, and 71.13±4.11 years, respectively. There was no statistically significant difference in age between the groups, showing comparability in terms of age (p>0.05).

**Table 1:** Distribution of age among the groups

Age (years)	Group A		Group B		Group C	
	No of patients (n)	%	No of patients (n)	%	No of patients (n)	%
65-70	15	50.00%	17	56.67%	16	53.33%
71-75	5	16.67%	8	26.67%	8	26.67%
76-80	9	30.00%	5	16.67%	6	20.00%
81-85	1	3.33%	0	0.00%	0	0.00%
Total	30	100%	30	100%	30	100%
Mean±SD	72.30±5.75		70.90±4.53		71.13±4.11	
p-value	A vs B - 0.150		A vs C - 0.185		B vs C - 0.418	

Table 2 shows the mean duration of surgery among the groups. The mean values were comparable among the groups.

**Table 2:** Distribution of mean duration of surgery among the groups

Parameters	Group A	Group B		Group C			p-value	
	Mean±SD	Mean±SD		Mean±SD		A vs B	A vs C	B vs C
Duration of surgery (minutes)	54.13±2.32	54.87	±2.1	54.70	±2.88	0.102	0.202	0.399

During the intergroup comparison, the mean HR was higher in group A compared to groups B and C at 5, 10, and 15 minutes, respectively, and observed to be statistically significant (p<0.050). Thereafter mean HR values between the groups were statistically not significant. Between groups B and C, HR remained comparable at all time intervals (p>0.050) (Table 3).

**Table 3:** Comparison of heart rate among the groups

Heart rate (HR) (beats/minute)		0 minutes	5 minutes	10 minutes	15 minutes	20 minutes	25 minutes	30 minutes	40 minutes	50 minutes	60 minutes
Group A	Mean±SD	78.10±6.43	83.23±10.78	83.40±12.14	81.53±12.19	80.37±12.23	80.57±9.66	79.63±8.08	78.30±7.85	78.67±7.49	77.40±7.41
	p-value (vs 0 minute)	-	0.015	0.025	0.098	0.200	0.139	0.210	0.457	0.377	0.352
Group B	Mean±SD	78.97±6.39	76.93±8.85	76.53±9.45	76.30±9.57	76.70±9.47	77.40±8.85	78.10±8.35	79.07±7.66	79.60±7.2	80.37±6.86
	p-value (vs 0 minute)	-	0.028	0.027	0.022	0.036	0.087	0.192	0.455	0.229	0.045
Group C	Mean±SD	78.93±6.92	76.07±8.92	75.07±9.49	74.70±9.63	75.13±10.38	76.30±10.55	77.97±10.16	79.03±9.4	80.20±8.62	81.57±7.94
	p-value (vs 0 minute)	-	0.002	<0.001	0.002	0.013	0.064	0.283	0.475	0.204	0.039
p-value	A vs B	0.301	0.008	0.009	0.035	0.100	0.095	0.236	0.352	0.312	0.056
	A vs C	0.315	0.003	0.002	0.010	0.040	0.054	0.242	0.372	0.233	0.020
	B vs C	0.492	0.353	0.275	0.261	0.272	0.332	0.478	0.494	0.385	0.267

Throughout the monitoring and reporting of data, MAP was consistently lower in group A compared to groups B and C and was statistically significant between the groups. However, the lowest variations in MAP were observed in group B. On intergroup comparison, MAP in group A was significantly less than in group B and group C (A vs B (p<0.001), A vs C (p<0.050)), while in group B MAP was significantly higher than in group C (p<0.050) (Table 4).

**Table 4:** Comparison of mean arterial pressure among the groups

Mean arterial pressure (mmHg)		Baseline 0 minute	5 minutes	10 minutes	15 minutes	20 minutes	25 minutes	30 minutes	40 minutes	50 minutes	60 minutes
Group A	Mean±SD	100.70 ±5.79	79.83±6.52	80.87±5.75	82.40±5.7	83.87±5.42	85.60±5.24	86.97±5.35	88.70±5.25	90.13±4.75	91.30±4.48
	p-value (vs 0 minute)	-	<0.001	<0.001	<0.001	<0.001	<0.001	<0.001	<0.001	<0.001	<0.001
Group B	Mean±SD	99.37±5.49	89.47±6.8	89.43±6.54	90.47±6.26	91.17±6.14	92.20±6.08	93.03±5.79	94.17±5.86	95.00±5.32	96.10±5.52
	p-value (vs 0 minute)	-	<0.001	<0.001	<0.001	<0.001	<0.001	<0.001	<0.001	<0.001	<0.001
Group C	Mean±SD	99.10±4.84	84.73±4.67	84.30±4.34	85.53±4.31	86.93±4.38	88.47±4.4	89.83±4.6	91.10±4.24	92.47±4.3	93.87±4.27
	p-value (vs 0 minute)	-	<0.001	<0.001	<0.001	<0.001	<0.001	<0.001	<0.001	<0.001	<0.001
p-value	A vs B	0.182	<0.001	<0.001	<0.001	<0.001	<0.001	<0.001	<0.001	<0.001	<0.001
	A vs C	0.125	<0.001	0.006	0.010	0.010	0.013	0.015	0.028	0.025	0.013
	B vs C	0.421	0.001	<0.001	<0.001	0.002	0.004	0.011	0.012	0.023	0.043

In the present study, the time of onset of sensory block was shorter in group A (3.08±11.57 minutes) compared to group B (11.57±1.48 minutes) and group C (5.47±1.25 minutes). During intergroup comparison, mean values were statistically significant between the groups (p<0.001). The time taken to reach the T10 sensory level was shorter in group A (6.13±0.9) followed by group C (15.53±1.5) and longer in group B (11.30±1.39 minutes). During intergroup comparison, mean values were statistically significant between the groups (p<0.001). The time taken to reach the complete motor block was fastest in group A followed by group C and longer in group B. During the intergroup comparisons, the mean values were found to be statistically significant (p<0.05). The patient satisfaction score was higher in group C (6.33±0.76) followed by group B (6.13±0.82) and least in group A (5.80±0.81). During intergroup comparison, mean values were significant between the groups while satisfaction scores were comparable between group A and group C (p>0.05) (Table 5).

**Table 5:** Comparison of various parameters among the groups

Parameters	Group A		Group B		Group C		p-value		
	Mean	±SD	mean	±SD	Mean	±SD	A vs B	A vs C	B vs C
Onset of sensory block (minutes)	3.08	±0.64	11.57	±1.48	5.47	±1.25	<0.001	<0.001	<0.001
Time to reach sensory block up to dermatome level T10 (minutes)	6.13	±0.9	15.53	±1.5	11.30	±1.39	<0.001	<0.001	<0.001
Time to reach motor block (minutes)	8.08	±1	20.33	±1.86	15.53	±1.31	<0.001	<0.001	<0.001
Patients satisfaction score	5.80	±0.81	6.13	±0.82	6.33	±0.76	0.059	0.005	0.165

Table 6 shows the comparison of complications among the groups. In the present study, the incidence of hypotension was reported in 26.67%, 13.33%, and 16.67% in groups A, B, and C, respectively. It was the most common complication that required a single dose of vasopressor and was comparable among the groups. Bradycardia was reported in 6.67% and 3.33% of patients of group A and group C but none of the patients in group B had episodes of bradycardia. Other complications included nausea and shivering which was more in group A as compared with group B and C remained comparable among the groups.

**Table 6:** Comparison of complications among the groups

Complication	Group A		Group B		Group C		p-value
	No. of patients	%	No. of patients	%	No. of patients	%	
Bradycardia	2	6.67%	0	0.00%	1	3.33%	0.355
Hypotension	8	26.67%	4	13.33%	5	16.67%	0.390
Nausea	4	13.33%	1	3.33%	2	6.67%	0.338
Shivering	4	13.33%	0	0.00%	0	0.00%	0.015

## Discussion

In our study distribution of age and duration of surgery were comparable among the groups (p>0.05). HR changes were maximum with group A compared to groups B and C, while it was least for group B and moderate for group C. The rise in HR was more in group A compared to B and C. The mean HR values remained comparable between groups B and C during the entire period of study. Mutahar et al. [13] conducted a prospective randomized study to compare the hemodynamic changes between the spinal block and SCSE for lower limb surgery and reported that there was a rise in pulse rate in the spinal group while stable hemodynamic parameters reported with sequential combined spinal epidural block similar to our study. Similar observations were also been reported during a randomized clinical study conducted by Sundar et al. [14] to compare the epidural and SCSE and found that mean values of pulse rate were comparable between the groups (p>0.05). In the present study, the fall in MAP was reported maximum under spinal block compared to epidural and SCSE, while the changes in MAP were least observed in the epidural group. However, mean values were statistically significant among all three groups (p<0.05). Similar observations were also reported by Mutahar et al. [13] Another study done by Jindal et al. [15] in 2007 observed better hemodynamic stability in the epidural group compared to general anesthesia and spinal block similar to our findings. However, the mean values were noted to be statistically significant between spinal and epidural groups (p<0.001). In the present study, mean Spo_2_ levels in different groups were comparable during all time intervals (p>0.050). During intergroup comparisons, mean values remained comparable between the groups (p>0.050). This is consistent with the study done by Jindal et al. [15]. Another randomized clinical study conducted by Sundar et al. [14] reported that mean RR changes and Spo_2_ values during anesthesia were comparable between both CSE and epidural groups (p>0.05).

In our study, it was observed that the onset of sensory and motor block was fastest in group A compared to C and B, while it was slowest for group B. Similar to our study, Patel et al. in 2017 [16] reported that the mean onset of sensory block was achieved earlier in the spinal compared to the SCSE group, but the mean values were comparable between the groups (p>0.05). The probable reason could be the use of fentanyl as an adjuvant in both groups. Another study conducted by Bhattacharya et al. [12] observed that the onset of sensory block was longer in the SCSE group compared to the spinal group and the mean values were not significant between the groups (p>0.050). In concordance with our study, Holmstrom et al. [17] concluded that the time interval between onset and T10 level sensory block differed among the groups and it was least for the spinal group and longest for an epidural group similar to our study. Patel et al. in 2017 [16] observed that patients in the spinal group observed grade III motor block (66.7%) and grade II block (33.3%), while in the SCSE group, grade III motor blockade was reported in 56.6% of patients and grade II blockade in 43.4% of patients. Similarly in our study, the profound motor block was achieved in the spinal block compared to SCSE. Sundar et al. [14] concluded that motor blockade (grade 3) was achieved in patients with combined spinal epidural compared to the epidural group. The study also showed that patient satisfaction score was higher for SCSE compared to epidural and spinal while it was least for the spinal group.

The frequency of adverse effects (hypotension, bradycardia, nausea, vomiting, shivering) was more in group A compared to groups B and C. Similar to our study more incidences of hypotension and bradycardia in the spinal group compared to the SCSE group were observed by Patel et al. [16] and Bhattacharya et al. [12]. Mutahar et al. in 2018 [13] also found more incidences of nausea and vomiting in the spinal group than in the SCSE group and similar incidences of hypotension in both groups. The incidence of bradycardia and hypotension was comparable (p>0.05) between the groups. None of the patients in our study developed post-dural puncture headache, transient neurological symptoms, epidural hematoma, nerve injury, or total or high spinal.

Limitations

These findings were based on a single-center study; however, to be more conclusive, they can be conducted at other centers as well. The advantage of SCSE with low doses of local anesthetics can be attempted on ASA Grades 3 and 4 patients with less hypotension when compared to a single-shot spinal with a large volume of the drug. Further research is advised in lower abdominal surgeries with various sensory levels as a result of the study being limited to orthopedic procedures. Other limitations include the involvement of different anesthetists and surgeons.

## Conclusions

A sequential combined spinal epidural block is found to be a better superior alternative to epidural block and spinal anesthesia, which combines the advantages of spinal and epidural blocks while minimizing their drawbacks. SCSE decreases the doses of local anesthetics required compared to the epidural for the desired level of block. SCSE assures hemodynamic stability more than spinal. SCSE seems to have a promising future in regional blocks.
